# Sex-specific mortality forecasting for UK countries: a coherent approach

**DOI:** 10.1007/s13385-017-0164-0

**Published:** 2018-02-02

**Authors:** Ree Yongqing Chen, Pietro Millossovich

**Affiliations:** 1 Strategy Consulting Team, NMG Financial Services Consulting Ltd, London, W1T 1JU UK; 2 Faculty of Actuarial Science and Insurance, Cass Business, School City University London, 106 Bunhill Row, London, EC1Y 8TZ UK; 3 DEAMS, University of Trieste, Via dell’Università 1, 34127 Trieste, Italy

**Keywords:** Mortality projection, Lee-Carter, Common factor, Coherent forecast, Cohort term

## Abstract

This paper introduces a gender specific model for the joint mortality projection of three countries (England and Wales combined, Scotland, and Northern Ireland) of the United Kingdom. The model, called 2-tier Augmented Common Factor model, extends the classical Lee and Carter [26] and Li and Lee [32] models, with a common time factor for the whole UK population, a sex specific period factor for males and females, and a specific time factor for each country within each gender. As death counts in each subpopulation are modelled directly, a Poisson framework is used. Our results show that the 2-tier ACF model improves the in-sample fitting compared to the use of independent LC models for each subpopulation or of independent Li and Lee models for each couple of genders within each country. Mortality projections also show that the 2-tier ACF model produces coherent forecasts for the two genders within each country and different countries within each gender, thus avoiding the divergence issues arising when independent projections are used. The 2-tier ACF is further extended to include a cohort term to take into account the faster improvements of the UK ‘golden generation’.

## Introduction

The last three decades have witnessed tremendous developments in the area of mortality modelling and forecasting, beginning with the Lee-Carter (LC) proposed in [26]. This pioneering paper rapidly gained popularity and credit due to its simplicity and ability to capture most of the variation in mortality rates. Over time, various extensions and variants of the basic LC model have been put forward, see for instance [2, 27, 35] and [5, 16] for a review and comparison. All these models focus on a single population. When they are applied independently in modelling multiple related subpopulations with similar demographic trends, they would generally lead to divergent forecasts.

Diverging trends over time for closely related subpopulations is usually not a desirable outcome. For example, due to genetic and biological reasons, male mortality rates have constantly been higher than female rates, see [23]. However, if male mortality improvements are faster than female ones and the two genders are projected independently, the model may forecast male mortality rates eventually lower than females. As noted in [Section 5.3,[8]], independent projection methodologies have to be adjusted in order to avoid divergence issues. It is also intuitively true that the mortality of populations that are geographically close or otherwise related is driven by a common set of factors such as social-economic conditions, health and care system, and the general environment. Therefore, non-divergent or ‘coherent’ models are sought to address the issue of divergence. The augmented common factor model (ACF) of [32] is an extension of the LC model and is an important step in producing a model that captures both the short-term divergence and long-term coherence among related populations (subpopulations). The ACF model, which we may also call *1-tier ACF*, uses a common factor to depict the long-term overall trend of the total population, with additional specific factors included to capture the short-term discrepancy from the common trend for each subpopulation. Several mortality models for multiple populations have been proposed in the last decade, see for instance [6, 11–13, 21, 22, 24, 28–30, 38, 39, 41]. See also [10, 14] for a review and comparison. However, most of the multi-population models introduced so far, including the ACF, have focused on achieving consistent forecasts among populations differentiated according to a single dimension - either gender or geographical difference, but not both.

In the UK, apart from age and gender being the traditional differentiating mortality factors, the social-economic differences among three countries (England & Wales combined, Scotland, and Northern Ireland) have led to notably different mortality trends, at least in the short-term. The aim of this paper is to introduce an extension to the ACF model, which we call *2-tier ACF*, where a common factor models the trend for the aggregated UK population, a sex specific factor captures the discrepancy between each gender and the total population, and a country/sex specific factor captures the discrepancy of a gender in a specific country from the overall trend of that gender. This specification ensures to achieve coherence of forecasts in both dimensions - mortality by gender within each country and mortality by country within each gender. The 2-tier ACF model is then further extended to include a gender-specific cohort term (*2-tier ACFC*), allowing for the fact that UK mortality experience in the past century cannot be explained by age and period factors only but requires terms depending on the year of birth, see [40].

The contribution of this paper is twofold. On one hand, we aim at introducing a model that, as described above, guarantees consistency across several dimensions, gender and country. On the other hand, we apply this model to the mortality experience of six subpopulations of the UK, consisting of two genders and three countries within each gender, England and Wales combined, Scotland and Northern Ireland. We use data from the Human Mortality Database for the period between 1975 and 2011 and project mortality rates up to the year 2050. The results from the 2-tier ACF and 2-tier ACFC are compared with the Lee-Carter model independently applied to each of the six subpopulations and the 1-tier ACF model applied independently to each couple of gender based populations within each country of UK. The fitting period is chosen so as to make sure that the period index of the common factor is reasonably linear. Due to the high volatility of mortality rates at the very old ages, we have excluded ages above 100 from the analysis.

## Forecasting models

### Lee-Carter and augmented common factor models

The Lee-Carter model [26] is defined below. Letting $m_{x,t}$ be the central rate of mortality at age *x* and time *t*, the LC model assumes that

1 $$\begin{aligned} \log m_{x,t}=a_x+b_x \kappa _t+ \epsilon _{x,t}, \end{aligned}$$

where $a_x$ represents the level of mortality at age *x*, $\kappa _t$ is an index of the mortality level at time *t*, $b_x$ represents the relative speed of mortality decrease at age *x*, and $\epsilon _{x,t}$ is an error term that is Gaussian distributed with mean zero and variance $\sigma ^2_\epsilon$.

The augmented common factor (ACF) model, also known as 1-tier ACF in this study, was originally introduced by [32]. It specifies the central rate of mortality $m_{x,t,i}$ at age *x*, time *t* for gender *i* ($i=f,\,m$), as

2 $$\begin{aligned} \log m_{x,t,i}=a_{x,i}+B_x K_t+ b_{x,i} \kappa _{t,i}+\epsilon _{x,t,i}, \end{aligned}$$

where $B_x K_t$ is the common factor for the aggregated population including both genders, $b_{x,i} \kappa _{t,i}$ is the sex-specific factor for gender *i*, and $\epsilon _{x,t,i}$ is the normally distributed error term. The term $K_t$ is designed to capture the overall time trend of the aggregated population, while $B_x$ measures the sensitivity to decrease in mortality at age *x*. The fact that subpopulations share the same component $B_x K_t$ is a necessary and sufficient condition in order to avoid divergence in central forecast of subpopulations, see [11]. Similarly, $\kappa _{t,i}$ is the mortality time index of a specific gender, and $b_{x,i}$ is the corresponding age sensitivity measure. The component $b_{x,i} \kappa _{t,i}$ hence captures the trend in mortality of the specific gender *i* on top of the overall trend of the aggregated population.

### 2-tier augmented common factor model

In this section, we introduce a new two-tier extension to the ACF model including a second additional factor for each specific country within each gender, resulting in a joint double-layer model for different sex and countries. We call this model the 2-tier Augmented Common Factor model (2-tier ACF).

Instead of assuming that errors are normally distributed and homoscedastic as in the original LC and ACF model, here, following [3], we model death counts directly as Poisson variables. Denote by $D_{x,t,i,j}$, $E_{x,t,i,j}$ and $m_{x,t,i,j}$ respectively the death counts, central exposure and central mortality rates at age *x*, time *t*, for the *i*-th gender and *j*-th country. The 2-tier ACF model is specified as follows:

3 $$\begin{aligned} D_{x,t,i,j}&\sim {\text {Poisson}}(E_{x,t,i,j}m_{x,t,i,j}),\end{aligned}$$

4 $$\begin{aligned} \log m_{x,t,i,j}&=a_{x,i,j}+B_x K_t+b_{x,i} \kappa _{t,i}+b_{x,i,j} \kappa _{t,i,j}. \end{aligned}$$

As in the ACF model, $B_x K_t$ is the common factor for the population aggregated across gender and countries while $b_{x,i} \kappa _{t,i}$ is the sex-specific factor for gender *i*. The factor $\kappa _{t,i,j}$ captures the mortality index of country *j* and gender *i* on top of the combined trend allowed for by $K_t$ and $\kappa _{t,i}$, while $b_{x,i,j}$ is the corresponding sensitivity at age *x*.

To ensure the identifiability of the model, we restrain parameters by imposing the following constraints:

5 $$\begin{aligned}& \sum _x B_x =1,\,\sum _t K_t =0,\nonumber \\&\sum _x b_{x,i}=1,\,\sum _t \kappa _{t,i}=0 \quad \text { for \quad all} \quad i,\nonumber \\& \sum _x b_{x,i,j}=1,\,\sum _t \kappa _{t,i,j}=0 \quad \text { for \quad all} \quad i \quad \text {and}\quad j. \end{aligned}$$

Once the different period terms have been estimated, they are modelled as observations of time series according to the following specification. As in the ACF model, the common factor time index $K_t$ is assumed to follow a random walk with drift,

6 $$\begin{aligned} K_t=K_{t-1}+d+z_t, \end{aligned}$$

where $z_t$ is a white noise process. Following [32], the period terms $\kappa _{t,i}$ for the two genders are modelled as weakly stationary, AR(1) time series,

7 $$\begin{aligned} \kappa _{t,i}=\alpha _{0,i}+\alpha _{1,i} \kappa _{t-1,i}+z_{t,i}, \end{aligned}$$

where the error terms $z_{t,i},\,i=m,f,$ are independent white noise processes that are independent of $z_t$, and $|\alpha _{1,i}|<1$ for $i=m,f$. Finally, the processes $\kappa _{t,i,j}$ are extrapolated by assuming again that they follow weakly stationary AR(1) time series,

8 $$\begin{aligned} \kappa _{t,i,j}=\alpha _{0,i,j}+\alpha _{1,i,j} \kappa _{t-1,i,j}+z_{t,i,j}, \end{aligned}$$

where the error terms $z_{t,i,j}$ are independent white noise processes that are independent of $z_t,\,z_{t,f}$ and $z_{t,m}$, and $|\alpha _{1,i,j}|<1$ for all *i*, *j*. Although there is no reason to exclude the possibility of fitting higher order ARIMA models for $K_t,\,\kappa _{t,i}$ and $\kappa _{t,i,j}$, for the sake of simplicity we choose to remain consistent with the prevailing literature and use a random walk with drift and an AR(1) time series. Also, the independence assumption among all mortality indices when extrapolating their future values could be relaxed, see the discussion in Section 4.2. The precise details of the maximum likelihood estimation algorithm used to fit the 2-tier ACF are given in the Appendix 1, but, on a high level, the algorithm follows the three major stages:

- fit $$\widehat{a}_{x,i,j}+\widehat{B}_x \widehat{K}_t$$;
- conditional on that, fit $\widehat{b}_{x,i} \widehat{k}_{t,i}$;
- conditional on the previous two stages, fit $\widehat{b}_{x,i,j} \widehat{k}_{t,i,j}$.

As an alternative to this estimation strategy based on successive stages, a maximum likelihood approach could be pursued where all relevant parameters, age and period terms, are fitted in a single step as in [10, 14]. Nonetheless, we prefer the former approach as it embodies the philosophy of the 2-tier ACF approach: each bilinear component is fitted in a way that best explains the overall trend of an aggregated population, leaving any trends particular to a subpopulation to the successive stage of the model fitting, See section 4.1 for an expanded discussion. As a by-product, introducing a hierarchy between bilinear terms overcomes the well-known identification issues arising in models spanning several period terms such as the Renshaw and Haberman two-term model (LC2), as analysed in [19]. The identifiability issues in multi-population models under a single step maximum likelihood approach have been thoroughly discussed in [14].

### 2-tier augmented common factor model with cohort extension

The 2-tier ACF model does not take into account the cohort effect - the mortality experience does not only depend on the calendar year, but is also related to the year of birth. In the UK, the cohort effect has a narrower meaning and it refers to the more rapid improvement in mortality experienced by the golden generation born between 1925 and 1945, see [40]. Here we introduce a simple cohort extensions to the 2-tier ACF model, called 2-tier augmented common factor model with cohort (2-tier ACFC), and defined by replacing (4) with:

9 $$\begin{aligned} \log m_{x,t,i,j}=a_{x,i,j}+B_x K_t+ b_{x,i} \kappa _{t,i}+b_{x,i,j} \kappa _{t,i,j}+g_{t-x,i}. \end{aligned}$$

The factors $g_{t-x,i}$ are gender-specific cohort term shared by all subpopulations within the same gender *i*. The cohort term is specified for each gender but not for each country, as the cohort effect differs between the two genders and is much more prominent in the residual plots for the whole UK than for each individual country. This choice of cohort extension will be further discussed in Section 4. The remaining parameters have the same meaning as in the 2-tier ACF model defined in Section 2.2. To avoid divergence between the two genders over time, $g_{t-x,i}$ should be extrapolated as a mean-reverting time series. Consistently with the modelling of the different period terms, an AR(1) process is used here for simplicity:

$$\begin{aligned} g_{h,i} = \beta _{0,i}+\beta _{1,i} g_{h-1,i}+w_{h,i}, \end{aligned}$$

where $h=t-x$, $w_{h,i},\,i=m,f$ are independent white noise processes that are independent of $z_t,\,z_{t,i},\,z_{t,i,j}$ for all *i*, *j*, and $|\beta _{1,i}|<1$ for $i=m,f$. To guarantee the identifiability of the model, the constraint $\sum _{h=t-x} g_{h,i}=0$ for all *i* is added to those already stated in (5). In the 2-tier ACFC model, the term $g_{t-x,i}$ should be fitted prior to fitting $b_{x,i,j} \kappa _{t,i,j}$, but after fitting the bilinear terms $B_x K_t$ and $b_{x,i}\,\kappa _{t,i}$, because $g_{t-x,i}$ is part of the common trend of gender *i* at the aggregated national level, and this aligns better with the principle behind the 2-tier ACF model that common factors are prioritised, before fitting any subpopulation specific factor.

## Comparison of the models

In this section we focus on the comparison, using fitting metrics, residual plots and long-term projection results, of the following four models fitted to the six subpopulations of the UK for the period 1975-2011 and forecasted to 2050:

- the LC model (1) applied to each of the six subpopulations independently;^1^
- the 1-tier ACF model (2) applied to each of the three couples of gender specific subpopulations within each country independently;^2^
- the 2-tier ACF with a common factor for the total UK population, a gender specific factor, and a gender-country specific factor;
- the 2-tier ACFC model with a gender-specific cohort extension on top of the 2-tier ACF model.

The independent 1-tier ACF model employed here follows the spirit of the P-division model introduced in [10], where the set of all subpopulations is partitioned into groups sharing some common characteristics. The mortality within group is then modelled using a common period term. In the present case, each subgroup is given by the two genders within each country. The common time factor is then complemented by adding an additional gender/country specific factor.

### Model fitting

The following metrics are examined in Table 1: Akaike information criterion (AIC), Bayes information criterion (BIC), Mean Absolute Percentage Error (MAPE) and Explanation Ratio (ER).^3^ The smaller the BIC, AIC and MAPE are, the higher the ER is, the better a model fits past experience.

**Table 1:** BIC, AIC, MAPE and ER of LC, ACF, 2-tier ACF and 2-tier ACFC. $f=$ females, $m=$ males

		Fitted model			
Metrics	Country	Lee-Carter	1-tier ACF	2-tier ACF	2-tier ACFC
BIC	Overall	214355	216459	205253	200153
AIC	Overall	202953	201786	190580	183300
MAPE	England and Wales (*f*)	0.05797	0.05787	0.05196	0.05045
	Scotland (*f*)	0.14843	0.14452	0.14229	0.14060
	Northern Ireland (*f*)	0.26665	0.25882	0.26243	0.26364
	England and Wales (*m*)	0.05546	0.05387	0.04437	0.04303
	Scotland (*m*)	0.12964	0.12711	0.12945	0.12357
	Northern Ireland (*m*)	0.21604	0.20522	0.20633	0.20672
	Overall	0.14570	0.14124	0.13872	0.13800
ER	England and Wales (*f*)	0.96856	0.96358	0.98057	0.99140
	Scotland (*f*)	0.91073	0.90641	0.91944	0.93077
	Northern Ireland (*f*)	0.85879	0.86411	0.85709	0.86290
	England and Wales (*m*)	0.98400	0.98488	0.98914	0.99555
	Scotland (*m*)	0.95979	0.96199	0.96229	0.96797
	Northern Ireland (*m*)	0.90242	0.91782	0.91493	0.91587
	Overall	0.97839	0.97731	0.98579	0.99369

All numbers are exact values rather than percentages

The AIC and BIC consistently rank the 2-tier ACFC, despite its relative complexity, above the other models. Independent specification of each gender in each country, or of each couple of genders within each country, does not seem to provide any substantial benefit compared to the aggregate modelling of all countries and genders. The addition a gender cohort term results in a further stark improvement of both indices.

It should be noted, from the values of MAPE and ER, that all models fit better to the mortality experience in England and Wales, less so to Scotland, and fit least well to Northern Ireland. This is due to the fact that populations with larger exposures have more stable historical mortality patterns and hence they are easier to fit using Poisson-type models that implicitly weigh populations according to their exposure. England and Wales is the largest population among the three countries; therefore the model best fits its experience, followed by Scotland and then Northern Ireland. It is clear from Table 1 that the 2-tier ACF fits better the historical experience than the independent LC or 1-tier ACF models according to both MAPE and ER, while the 2-tier ACFC further improves the model fitting, its extent varying from moderate to substantial depending on the country and gender. One notable exception is Northern Ireland, where the 1-tier ACF slightly outperform the other models, confirming nonetheless the need of country specific period terms common to both genders.

### Residual plots

In this section, we inspect the residual plots of the four models against cohorts to assess the models’ capacity in capturing systematic variations by cohort. The residual plots against age and calendar year are fairly similar among different models and are included in Appendix 1.

According to [28, 34], because the model fitting uses an over-dispersed Poisson distribution, the scaled deviance residuals are given by the equation

$$\begin{aligned} {{\mathrm{sgn}}}{(d_{x,t,i,j}-\widehat{d}_{x,t,i,j})} \sqrt{\frac{{\text {dev}}(x,t,i,j)}{\widehat{\phi }}}, \end{aligned}$$

where

$$\begin{aligned} {\text {dev}}(x,t,i,j)&=2\left( d_{x,t,i,j} \log \frac{d_{x,t,i,j}}{\widehat{d}_{x,t,i,j}}-d_{x,t,i,j}+\widehat{d}_{x,t,i,j}\right) ,\\ \widehat{\phi }&=\frac{\sum _{x,t,i,j}{\text {dev}}(x,t,i,j)}{n_d-n_p}. \end{aligned}$$

**Fig. 1:**
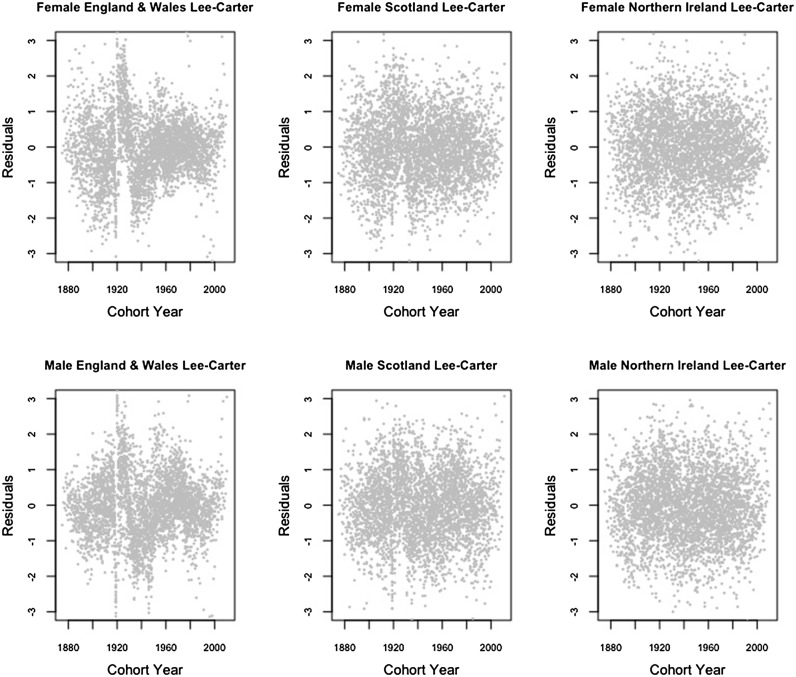
Residual plots by cohort of the LC model applied to the three countries and genders of UK

**Fig. 2:**
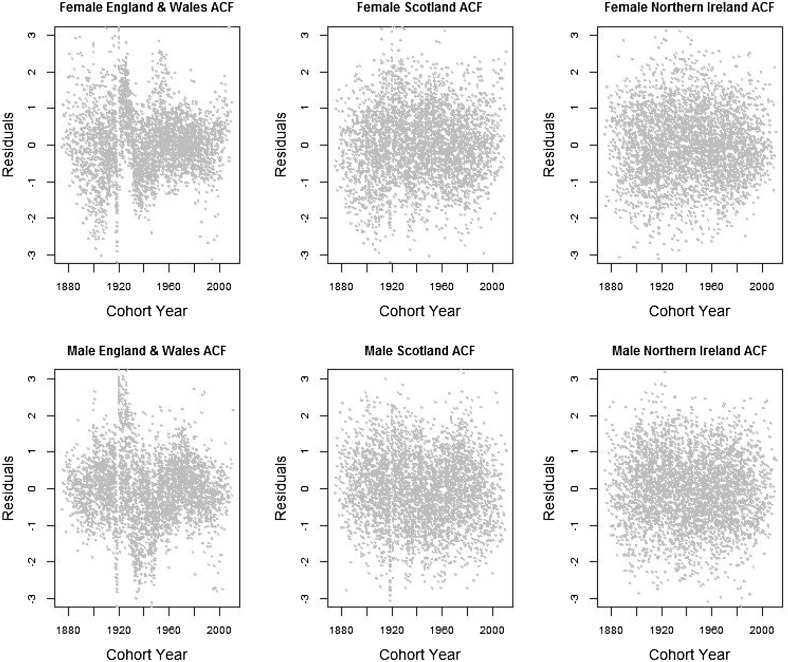
Residual plots by cohort of the 1-tier ACF model applied to each couple of genders within the three countries of UK

**Fig. 3:**
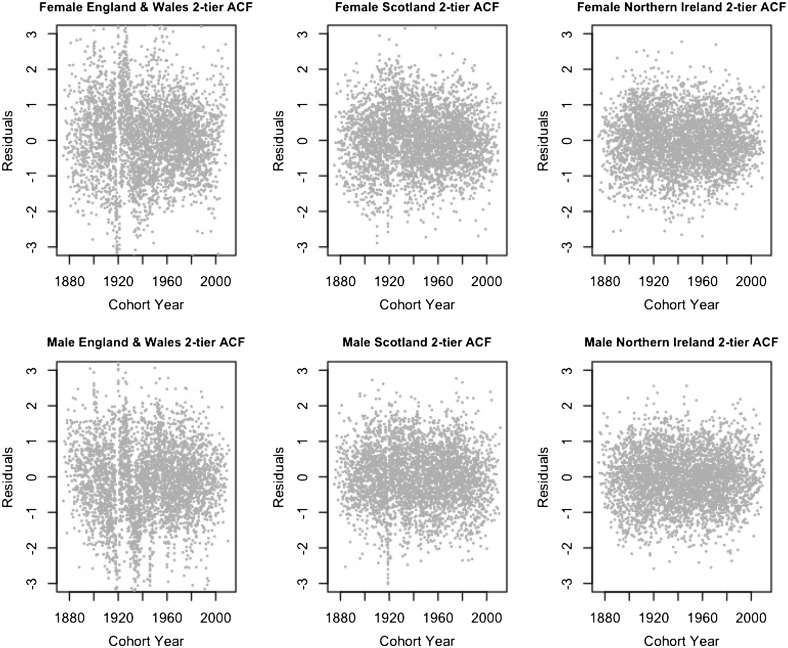
Residual plots by cohort of the 2-tier ACF model applied to the three countries of UK

**Fig. 4:**
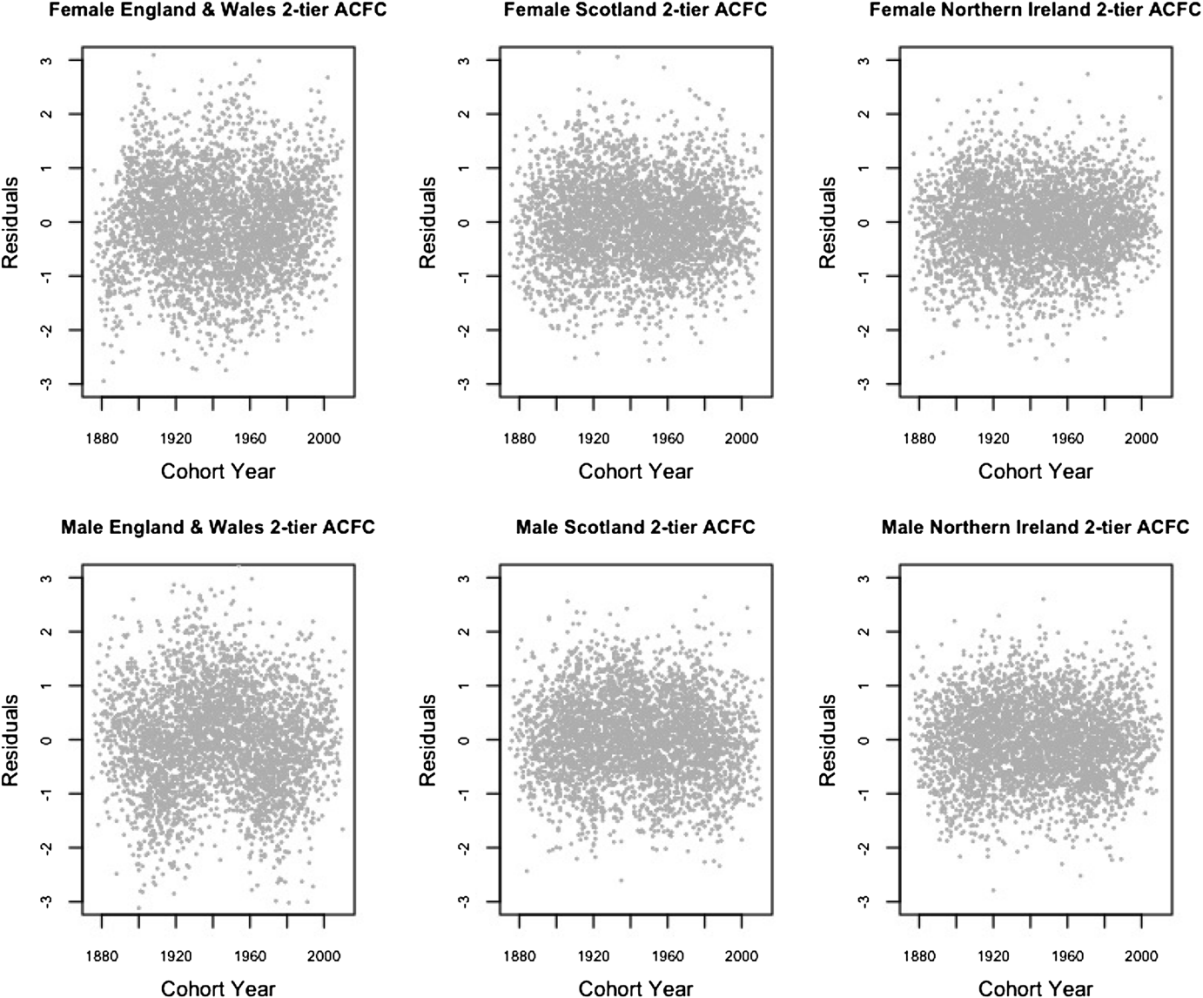
Residual plots by cohort of the 2-tier ACFC model applied to the three countries of UK

Figures 1, 2, 3 and 4 give the residual plots against cohort for all six subpopulations in the UK. For England and Wales and Scotland, there is a marked increase in the randomness of residuals from the independent LC or independent 1-tier ACF models (Figures 1, 2) to the 2-tier ACF model (Figure 3), and also from the 2-tier ACF model to the 2-tier ACFC model (Figure 4). This means that the 2-tier ACF model already captures some of the cohort effect internally due to the finely grained fitting of the bilinear terms, while the cohort term in the 2-tier ACFC further reduces the systematic pattern in the residual plots dramatically. However, in the 2-tier ACFC England and Wales male plot, some systematic pattern is still present, suggesting the potential inclusion of an age modulator for the England and Wales cohort term. For Northern Ireland, the cohort effect is not obvious even in the residual plots of the independent LC model, so when we look at the mortality rates of Northern Ireland on its own, the gender-specific cohort term could in principle be dropped.

### Long-term projection

In this section, the long-term projection behaviours of the models are compared, with a focus on the cross-age smoothness, coherence among countries and robustness of estimates in gender gaps. The independent LC, independent 1-tier ACF, 2-tier ACF and 2-tier ACFC, fitted to the period 1975-2011, are now used to project future mortality rates up to 2050.

**Fig. 5:**
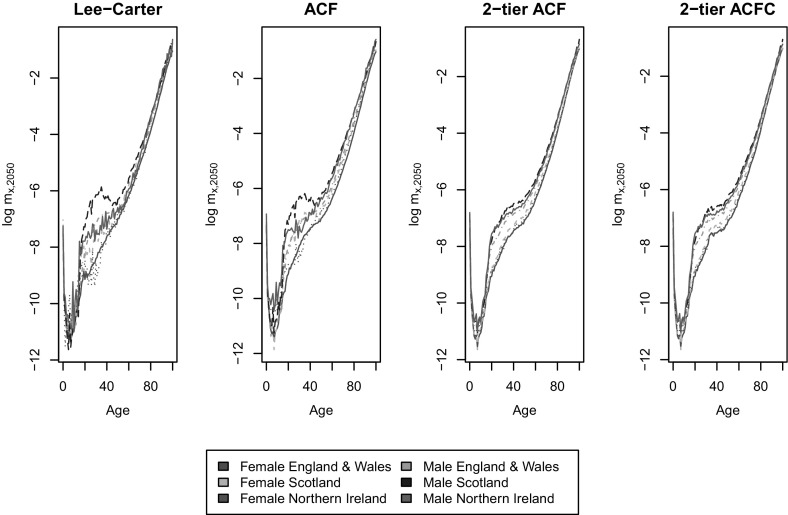
Projected central death rates ($\log$ scale) by age and country in 2050

**Fig. 6:**
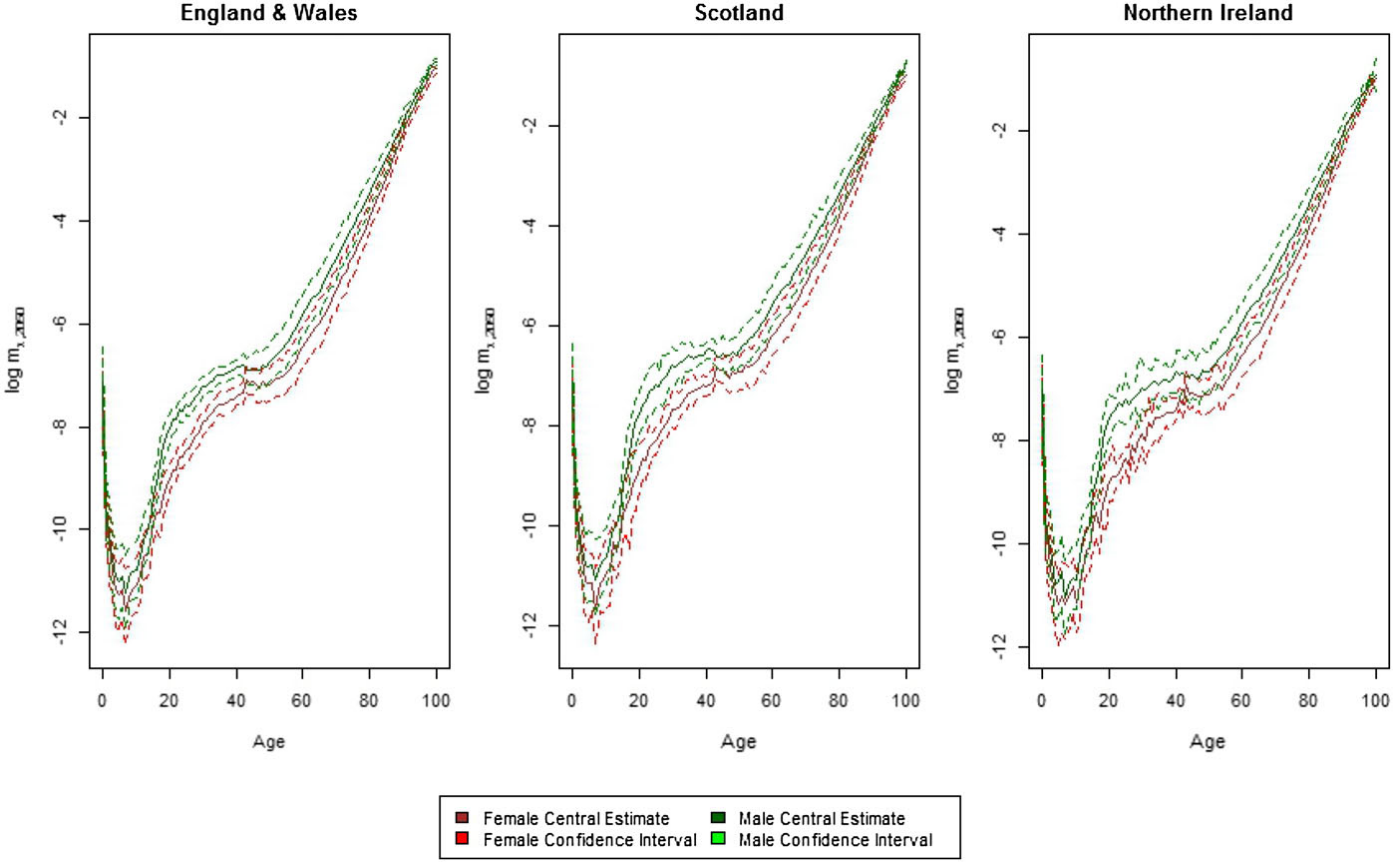
Projected central death rates ($\log$ scale) by age and country in 2050 for the 2-tier ACFC model and 95% confidence bounds

Figure 5 gives the central estimates of log-scale mortality rates by age in year 2050 in the four models. Firstly, the 2-tier ACF forecasts much smoother age to age mortality rates as compared to the independent LC and 1-tier ACF. The independent LC projection for Scotland male even shows decreasing mortality by age at around age 40. Lack of cross-age smoothness of the LC model has long been highlighted in research, see for instance [5], as it uses only one age modulator $b_x$ to measure the age sensitivity to mortality improvement for the specific subpopulation and assumes that it remains constant. Over time, small differences between nearby $b_x$ terms lead to large discrepancies in mortality forecasts between neighbouring ages, causing in turn lack of smoothness. The independent 1-tier ACF model, despite the presence of a common bilinear term, seems to be affected by the same issue. However, in the 2-tier ACF model, for each subpopulation the mortality improvement trend is decomposed into tiers - the common trend of total population, the trend of a specific gender, and the trend of the specific subpopulation. Overall, the more finely grained model produce an age pattern displaying smoother cross-age mortality improvement. The cohort factor in the 2-tier ACFC however, adds slightly more cross-age volatility to ages between 30 and 40 than the 2-tier ACF model, as now mortality at a specific age in a calendar year is also dependent on the variations from the year of birth. The difference, however, is negligible.

Secondly, Figure 5 shows that the LC method produces much larger differences among countries within each gender, and between different genders within each country, especially for the age range between 20 and 60. This is consistent with our expectation that independent extrapolations of different subpopulations under the LC method will produce divergent mortality rates for related populations, whereas the ACF framework partially avoids such issue. As pointed out by [6], under the ACF paradigm, the global improvement trend will dominate over time, due to the fact that the subpopulation-specific components are mean reverting. The 2-tier ACF further extends the ACF model, so that the projections for different countries are dominated by the common gender trend. In other words, this extension ensures that the ratios of different subpopulations of the same gender converge over time, because the trend of the gender as a whole dominates over the trend in the specific subpopulation. For subpopulations in countries *j* and *k* of the same gender *i*, the difference of age specific mortality (on a $\log$ scale) is given, from (4), by:

$$\begin{aligned} \log m_{x,t,i,j}-\log m_{x,t,i,k}=(a_{x,i,j}-a_{x,i,k})+(b_{x,i,j} \kappa _{t,i,j}-b_{x,i,k}\kappa _{t,i,k}). \end{aligned}$$

As $\kappa _{t,i,j}$ and $\kappa _{t,i,k}$ are mean reverting processes, it is clear that the mortality spread is a mean-reverting process too. Hence, the differences in mortality rates between countries are more constrained in the 2-tier ACF projection compared to the independent LC or even the independent 1-tier ACF. A similar remark applies to the 2-tier ACFC model.

Figure 6 isolates the projection of age-specific mortality rates according to the 2-tier ACFC model, together with confidence bounds. For England and Wales and Scotland, relatively narrow confidence intervals reflect the sizes of the corresponding populations. For young adult (age 20 to 40) the confidence regions of males and females are separated, implying that, even over a long horizon, mortality convergence between sexes will be observed only at young and old ages. For Northern Ireland, slightly wider confidence bounds are obtained as a consequence of its smaller population. In this country, the apparent lack of smoothness across age of the projection is put into the right perspective when comparing it with the corresponding projection in Figure 5 under the independent LC or 1-tier ACF model. The presence of period terms spanning the three countries helps in dramatically reducing the age-to-age variation of mortality rates forecast.

**Fig. 7:**
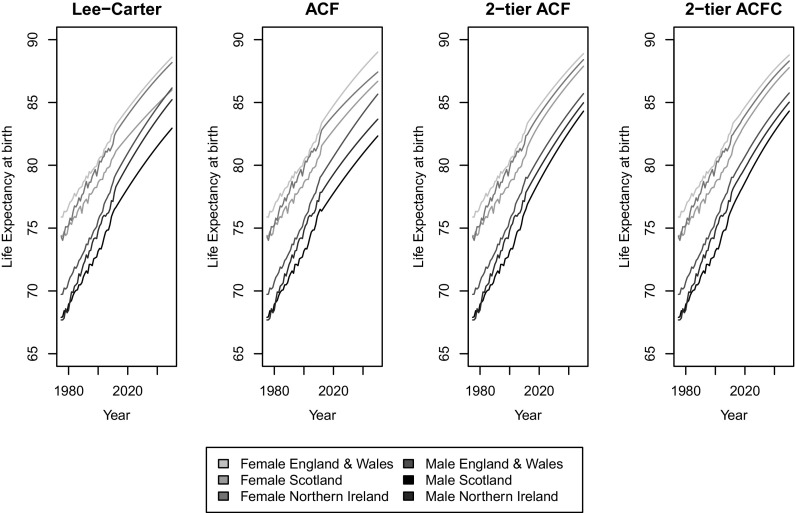
Life expectancy at birth by calendar year and country

Figure 7 shows the projected life expectancy at birth for all six subpopulations, using the four models. For the independent LC model, life expectancy forecasts are diverging. In particular, there is an increasing gap in life expectancy between Scotland and the rest of the UK for both genders. Not surprisingly, although to a lesser extent, this divergence is also observed for the independent 1-tier ACF model. As suggested by [33], the higher mortality experienced by Scotland before 1980 was most likely due to the deprivation and poverty linked to the industrial employment patterns. Since 1980, the cause of the higher mortality in Scotland is most likely related to the community disruption caused by deindustrialisation, which affected the West of Scotland more than the rest of UK. These essential historical factors may be continuing to the present day, implying lower life expectancy in Scotland as compared to the rest of the UK. However, it is difficult to justify an increasingly widening gap in mortality between (geographically, politically and socially) related countries in four decades time. Scotland is the only country so far providing free personal social care for those aged 65 or above, and has a level of health funding per head much higher than England. Latest research has also shown that the gap of health system performance indicators has narrowed between Scotland and rest of UK due to dramatic improvements in Scotland since 2010, see [1, 9]. Greater regional equality across the UK is an objective underlying all public policies, so it is reasonably expected that the gap within countries in the same gender should be narrowing down, as it can be observed in the 2-tier ACF (and 2-tier ACFC) model. There is no material difference between these two models in terms of long-term life expectancy projection, but a closer inspection shows that within each gender, the country gaps are closing at a slower pace if the cohort effect is considered.

**Fig. 8:**
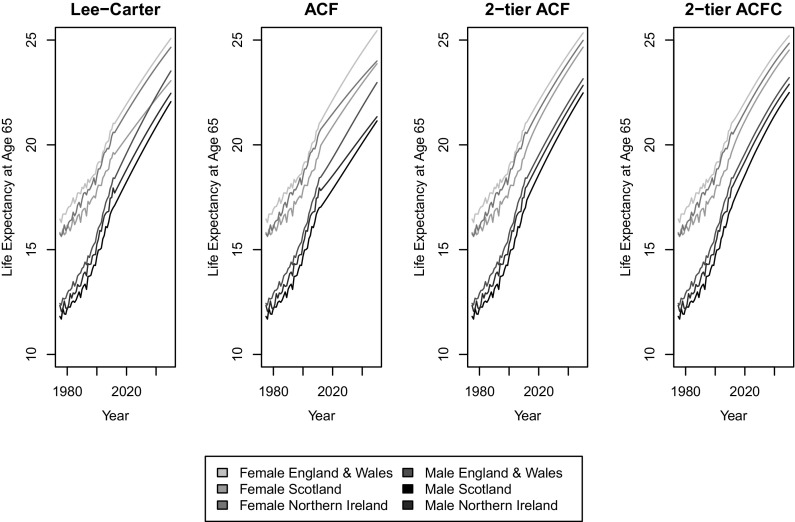
Life expectancy at age 65 by calendar year and country

The differences among the four models become more obvious in Figure 8, when life expectancy at retirement age 65 is projected. The independent LC model even forecasts an increasing gap between Northern Ireland and England and Wales for males, and the independent 1-tier ACF extends this undesirable pattern to females as well. Although Northern Ireland’s higher rate of suicide, maternal and infant conditions and cancers have historically contributed to the male life expectancy gap, since 1980–82 Northern Ireland’s life expectancy has been improving at a faster pace than England and Wales, see [25]. A slowly narrowing gap allowing for short-term disparities, as forecasted by the 2-tier ACFC, provides a much more reasonable outlook.

The 2-tier ACFC (or 2-tier ACF) model also ensures that the male-female expected mortality ratios (on a $\log$ scale) converge over time to long term limits that are constrained in a way that is appropriate when comparing related countries. In the 2-tier ACFC, for country *j*, the male-female mortality ratio on a $\log$ scale is given, from (9), by:

$$\begin{aligned} \log m_{x,t,m,j}-\log m_{x,t,f,j} = \, &(a_{x,m,j}-a_{x,f,j})+(b_{x,m} \kappa _{t,m}-b_{x,f} \kappa _{t,f})\\&+(g_{m,t-x}-g_{f,t-x})+(b_{x,m,j} \kappa _{t,m,j}-b_{x,f,j} \kappa _{t,f,j}). \end{aligned}$$

For each country, the male-female mortality ratio will share the common component $(b_{x,m} \kappa _{t,m}-b_{x,f} \kappa _{t,f})+(g_{m,t-x}-g_{f,t-x})$, which is reverting to a positive long term mean capturing the overall trend in gender differences for all territories. The component $b_{x,m,j} \kappa _{t,m,j}-b_{x,f,j} \kappa _{t,f,j}$ could possibly converge to a non-zero mean, but after fitting the overall trend and gender trends, $\kappa _{t,m,j}$ and $\kappa _{t,f,j}$ are normally best fitted by AR(1) processes with long term zero mean - the results actually show that male-female ratio of each country converge to the same positive limit over time.

**Fig. 9:**
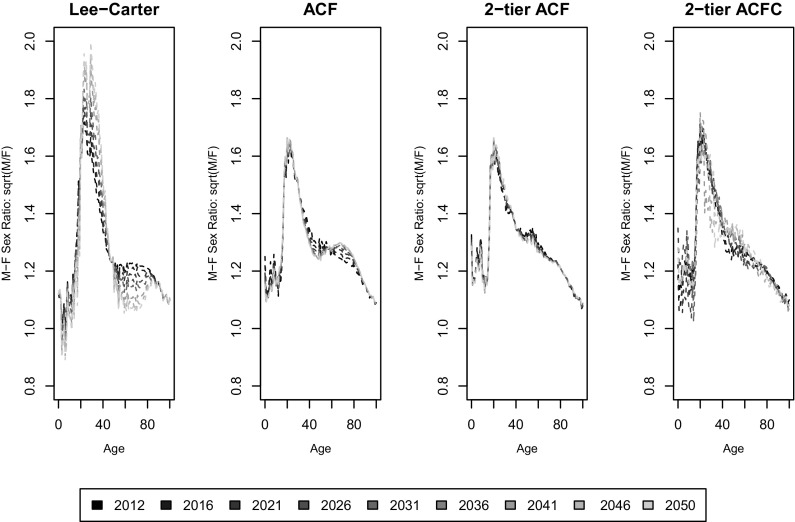
Male-female mortality ratio (square root scale) by age for a selection of projected years for England and Wales

**Fig. 10:**
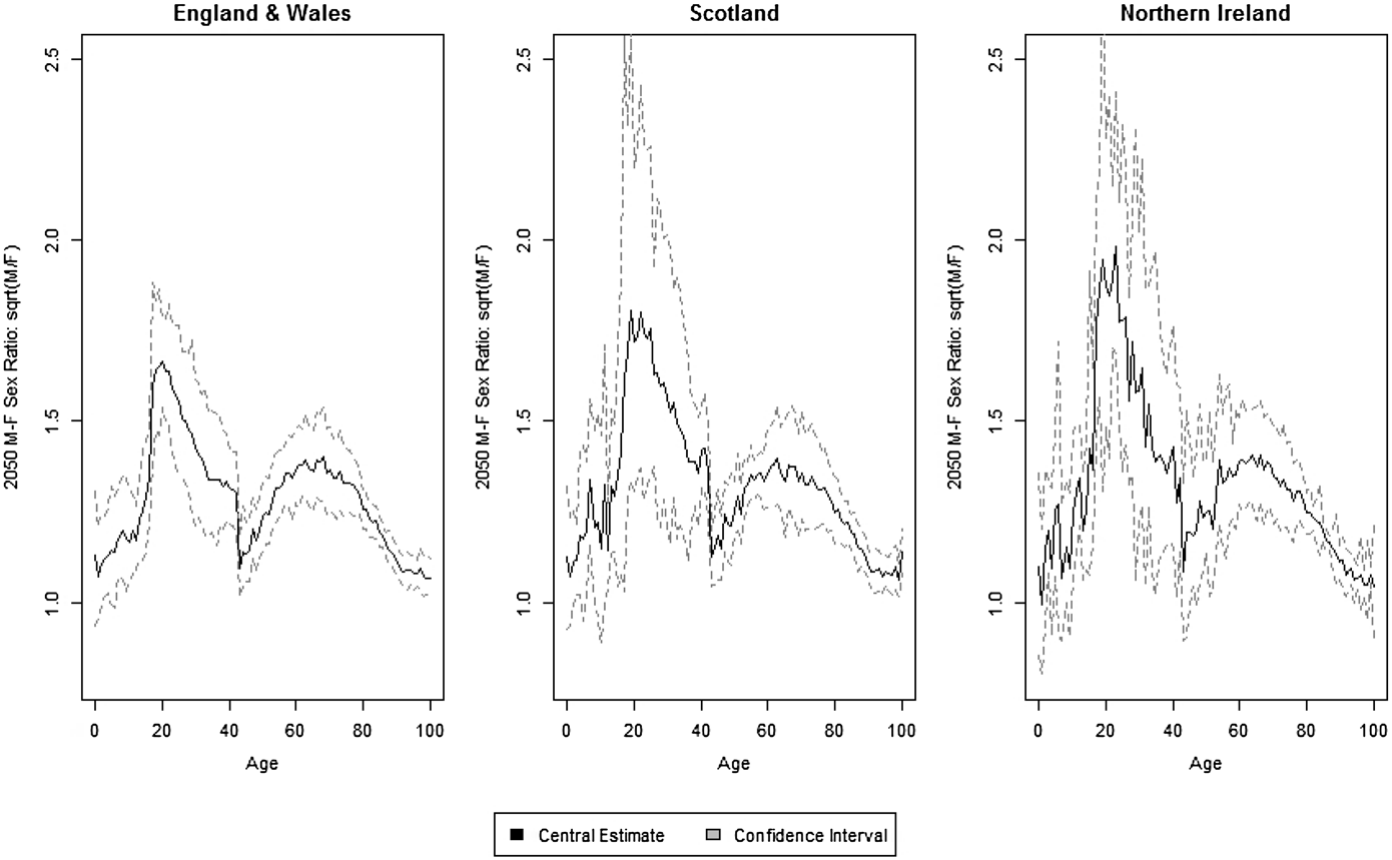
Male-female mortality ratio (square root scale) by age and country for a selection of projected years for the 2-tier ACFC model and 95% confidence bounds

In Figure 9, the male-female mortality ratio (on a square root scale) for England & Wales is plotted against age for a selection of years, and the results are in line with the understanding that sex differences in mortality are mainly contributed by the high mortality of very young and middle aged males, see [23]. It can be seen that for the LC projection, as also found by [21], at the very young ages, when the number of deaths is very small, undesirable projection outcomes of sex ratios less than 1 may occur. The coherent projections under the 1-tier and 2-tier ACF do not have such issues. The independent LC produce increasing sex ratios up to as high as 2 in 2050 for age groups between 30 and 50, again showing the undesirable features of divergence in long-term projections, while sex ratios from the 2-tier ACF model remain stable and constrained. However, sex ratios in England and Wales follow a stable pattern for the successive 40 years under the 1-tier and 2-tier ACF model, which is unlikely to be true. Compared to the 2-tier ACF, projecting the cohort factor of each gender independently introduces some additional variation over the forecast years for the 2-tier ACFC, while keeping the sex ratios constrained in a stable and reasonable range. Figure 10 isolates the male-female mortality ratio (on a square root scale) for the 2-tier ACFC model for the three countries, together with confidence intervals. Again, the uncertainty around mortality ratios reflect the corresponding population sizes, with Northern Ireland dominating Scotland which in turn dominates England and Wales. It is remarkable that, for Northern Ireland, mortality for some young adult males is forecast to be as high as four times as the corresponding female mortality.

## Further discussions and concluding remarks

### Critical appraisal of the 2-tier ACFC model.

**Fig. 11:**
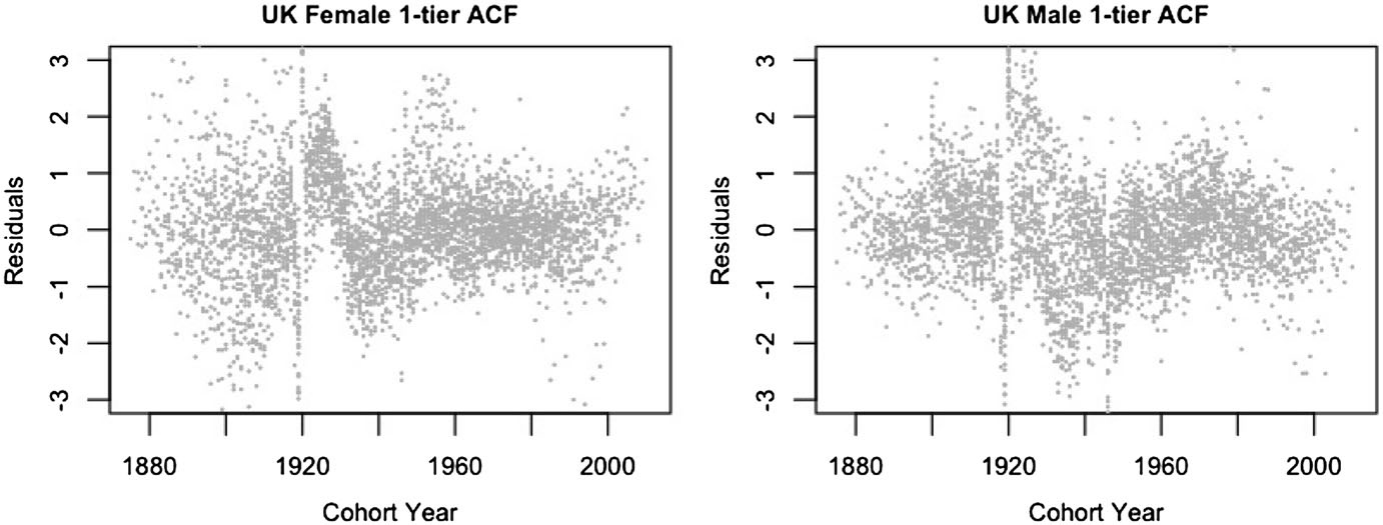
Residual plots by cohort of the 1-tier ACF for females and males for the three countries of UK combined

In (9), the cohort term is gender specific but not country specific. This is primarily driven by the finding that the cohort effect for males and females of the whole UK observed in the 1-tier ACF residuals (Figure 8) is much more significant than the cohort effect for each of the six subgroups under the 2-tier ACF (Figure 5). Also, in Figure 11, very distinctive cohort patterns for different genders can be seen. Therefore, we believe that the cohort trend is more significant on a gender specific level, and, in the 2-tier ACFC model, it should be fitted after estimating the term $a_{x,i,j}+B_x K_t$ but before fitting the term $b_{x,i,j} \kappa _{t,i,j}$.

This approach is analogous to that of [41] to fit cohort extensions of the Poisson Common Factor Model (PCFM) of [28], but is fundamentally different from the method proposed by [36] where, when extending the LC model to include a cohort term, the latter is fitted together with the period factor. However, the approach in [36] cannot be readily applied into the ACF framework, as the multiple bilinear components of the ACF are arranged in hierarchy, so that common trends are fitted prior to fitting individual subpopulation trends. Therefore, the term $g_{t-x,i}$ would have to be placed within this hierarchy and should be fitted after the term $a_{x,i,j}+B_x K_t$ but before the bilinear term $b_{x,i,j} \kappa _{t,i,j}$, for the model to make sense. This is another key feature of the 2-tier ACFC: including a cohort term still gives rise to a coherent forecast in terms of differences in mortality among subpopulations, because the common trend of the entire population is prioritised while the term $g_{t-x,i}$ is modelled as a stationary process. It may be argued that a common gender cohort factor $g_{t-x}$ could be fitted, together with the bilinear term $B_x K_t$, so as to maintain the coherence property. However, the residual plots from the 1-tier ACF suggests that cohort patterns do differ between different genders, which is consistent with the findings in [40].

The approach used in this paper also fits $b_{x,i} \kappa _{t,i}$ prior to fitting $g_{t-x,i}$, setting in this way the priority of period factors over cohort factors. This is consistent with the assumption that mortality depends more on the calendar year than on the year of birth when fitting the idiosyncratic trend for each gender. Some research findings, however, disagree with this assumption. In [37] it is suggested that, when fitting the mortality rates of the elderly population in the UK, the cohort effect is more prominent than the period effect. This may suggest alternative orderings when fitting the different components of the ACFC model - one might choose to fit the cohort factor $g_{t-x,i}$ prior to fitting any bilinear term $b_{x,i} \kappa _{t,i}$, or at least to jointly fit them in a single step when minimising the deviance function. [15] also suggest that the order of model fitting in age-period-cohort models makes a huge difference to parameter shapes. Further research may therefore be able to identify more elegant ways of including the cohort extensions within the 2-tier ACF hierarchy.

**Fig. 12:**
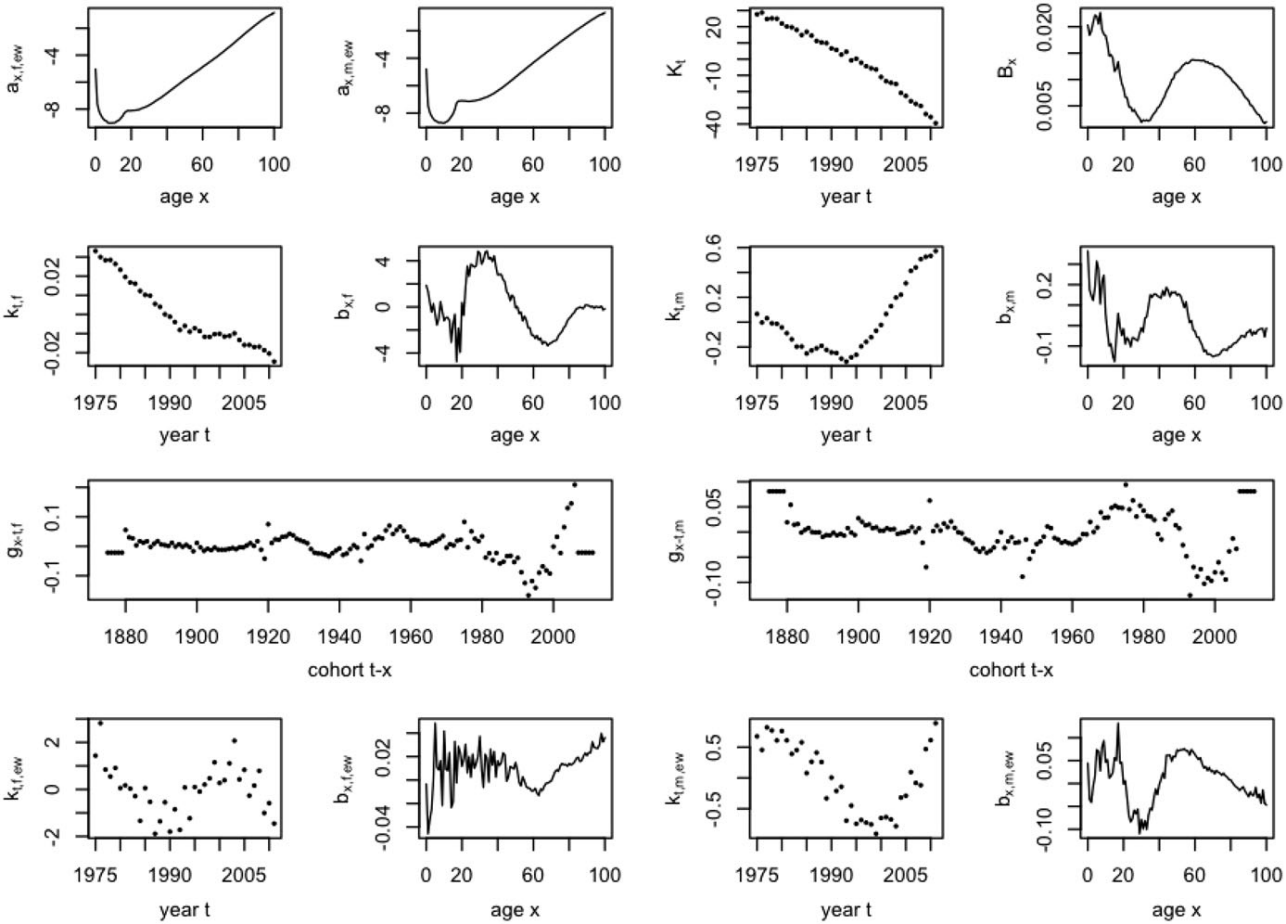
Plots of the 2-tier ACFC parameters $a_{x,i,j},\,B_x,\,K_t,\,b_{x,i},\,\kappa _{t,i},\,b_{x,i,j},\,\kappa _{t,i,j}$ and $g_{t-x,i}$ for England & Wales ($i=f$ or *m*, $j=EW$)

It should also be noted that it only makes sense to extrapolate $g_{t-x,i}$ as stationary process when $a_{x,i,j}+B_x K_t+b_{x,i} \kappa _{t,i}$ is prioritised in the fitting process, as it is the residuals after fitting these components that drive the shape of $g_{t-x,i}$. The plots of cohort factors produced by [41] are much more erratic compared to those in [36]. This is primarily because the PCFM, as used by [41], uses up to five sex-specific bilinear terms to capture the trends of a gender departing from the overall combined population, and if the whole PCFM model is fitted prior to fitting any cohort extension, the residuals used to fit such cohort term are already very erratic. However, since we impose that the term $g_{t-x,i}$ is fitted after the component $a_{x,i,j}+B_x K_t+b_{x,i} \kappa _{t,i}$ but before the term $b_{x,i,j} \kappa _{t,i,j}$, the cohort factor turns out to be less erratic (Figure 12) and easier to interpret. If the cohort factor shows a negative slope, it means that mortality in that cohort is improving at a faster pace than implied by the 1-tier ACF model. One can easily spot in Figure 12 the golden generation of those born between 1925 and 1945, especially for females, which is consistent with [40]. Another merit of the current approach is that it generally avoids the issues in the two steps method adopted by [36] that the fitting algorithm may not converge for certain combinations of data, parameters and identifiability constraints, which makes the cohort factor harder to interpret, as is pointed out by [20].

The cohort factor is sometimes modelled as a non-stationary (integrated) process, as by definition it should capture the structural changes in mortality patterns by cohort. However, because the approach taken here prioritises the model fitting of certain age and period terms, some cohort patterns may already be implicitly captured, due to the simple fact that cohort is merely age netted off the calendar year, and the cohort terms are intrinsically related to the prioritised age and period terms. Nevertheless, whether the residual cohort effect represented by $g_{t-x,i}$ in the ACFC models really represents structure trends in mortality and should be extrapolated into the future using a stationary process are areas involving a lot of subjective judgements. What we can conclude from the above analysis is that the cohort factors fitted under this method display reasonable trends over time and can be easily interpreted, although the pattern gets more erratic in later cohorts (namely after 1975); the cohort factors also improve the fitting of the model, evidenced by the lower BIC and AIC.

### Limitations of the 2-tier ACF/ACFC models.

Firstly, the method fundamentally belongs to the class of models described as ‘extrapolative’, so it can only capture trends well embedded in the historical data and lack the ability to project more up-to-date information such as medical progresses, environmental and social-economic changes such as, for example, the increasing female participation in the workforce, see [18].

Secondly, the 2-tier ACF/ACFC models are extensions of the LC model. A major issue of such class of models is that they neglect the existence of an age-time interaction. More specifically, rates of mortality change $b_x$, $b_{x,i}$, and $b_{x,i,j}$ are assumed to remain constant over time, whereas substantial age-time interactions have been identified in actual experience, see [27]. This results in the fact that the models tend to underestimate the life expectancy. In [7], a possible extension of the LC method accounting for the changing age sensitivity to mortality improvement by applying the LC method is proposed. This extension could be potentially applied to the 2-tier ACF/ACFC models to consider the evolving pattern of age modulating terms.

Another issue of the 2-tier ACF framework is that it assumes homogeneity at different levels. When the $B_x K_t$ term is fitted, homogeneity is assumed for all lives aged *x* in year *t*, but when $b_{x,i} \kappa _{t,i}$ is estimated, homogeneity is assumed for all lives aged *x* in year *t* with the same gender, and the assumption is further relaxed when the model is extended to the country dimension. It should be noted that homogeneity assumptions were embedded in the basic LC model, and methods to build in heterogeneity into the framework has been suggested by [31].

Throughout this research, we have proposed to fit, for simplicity purposes, an AR(1) or random walk to all the mortality period indices, instead of other higher order ARIMA models which may fit better past experience. Moreover, the mortality indices in the model have been extrapolated independently. Despite the fact that $\kappa _{t,i}$ and $\kappa _{t,i,j}$ may be correlated and a vector approach may further improve the model forecasting, see for instance [21], each period index in the ACF/ACFC framework represents a trend of a subpopulation that departs from the general trend of the aggregated population, justifying therefore the independent extrapolation used in the paper. Moreover, if a vector approach were considered, correlations among time indices would have to be estimated, compromising the simplicity of the model. Similarly, an AR(1) was chosen to the fit cohort terms in the 2-tier ACFC model, which are then extrapolated independently. Although historically females and males have displayed different cohort patterns in their mortality improvements, there could be interactions between the cohort effects of the two genders, since inevitably females and males born in the same year are exposed to similar social-economic context and healthcare facilities. Therefore, a more sensible approach may consist in fitting and extrapolating the cohort factors using a vector time series.

Most of the results considered in this paper are point estimates for future mortality rates. Further research should look into the statistical errors of estimates, which are primarily driven by standard errors of parameters in fitting the mortality time indices. The 2-tier ACF/ACFC model could be potentially extended to include more tiers to form coherent estimates in several dimensions, for instance taking into account regional inequalities within each country. However, further division within each sex and country means that the sample size of each subpopulation would be smaller and may produce less statistically significant results. Using a different perspective, one may wonder whether the role played by the two factors used to disaggregate mortality improvements, namely gender and country, could be interchanged, i.e. interpret *i* as the country index and *j* as gender index. This *reversed* 2-tier ACF model would then fit a common bilinear term, followed by a country specific term and finally a gender specific term within each country. In the current example based on three countries of UK, the two alternatives are bound to produce similar results, as both are rich enough to represent (implicitly or explicitly) sex differences between countries and country differences within each sex. The reverse ACF would only require one additional bilinear term. In a more general example where *I* countries were to be modelled, the direct 2-tier ACF based on gender first/country second will require $1+2(1+I)$ bilinear terms. The reversed 2-tier ACF based on country first/gender second will need $1+3I$ bilinear terms. As the number of countries *I* grows, the eventual benefit of adopting the reversed approach would be overshadowed by the increased number of parameters to be estimated.

The 2-tier ACFC model can be improved in several directions. In particular, the common age effect model introduced recently by [24] is worth mentioning. Unlike the common factor paradigm, under this approach different populations feature different period trends but share some of the corresponding age modulating parameters. The idea is that, while mortality improvements are free to vary between subgroups, the corresponding age specific changes will be in common between the same subgroups. This could be very convenient when some of the subgroups have small exposures, negatively affecting the properties of the corresponding age response term estimators. Inheriting the age terms from subgroups with larger size will provide a relief against this issue, adding up to the overall benefit coming from the reduction in the number of parameters. This approach has been taken up in [17] in the context of basis risk assessment in longevity transfers, where the mortality of (small) pension schemes relative to the national population needs to be assessed. In the application considered in this paper, the 2-tier ACFC could be complemented by letting some of the subgroups at gender/country level share the age response term with other subgroups. This could help, for instance, in reducing the lack of smoothness in some projections such as those of Northern Ireland as evidenced in Figures 6,10. A similar idea has been pursued in [14], where the Li and Lee model is simplified by restricting some of the age response terms, relative to the country specific or to the overall period effects, to be equal.

## Conclusions

We have extended the ACF model proposed by [32] to a 2-tier structure in order to model subpopulations of different genders and countries jointly and coherently. A Poisson structure similar to that in [28] is applied to introduce a robust statistical framework for testing the accuracy of model fitting. The 2-tier ACF model fits better the historical mortality experience of the six subpopulations than the independent LC model and the independent Li and Lee models applied to each couple of genders separately. For long-term projections, the 2-tier ACF model produces coherent results for both gender difference within each country and country differences within each gender. The 2-tier ACF model is also extended to the 2-tier ACFC by including a cohort factor, which further improves model fitting, removes significant patterns displayed in the plots of cohort residuals, and maintains the coherence property in long-term projection.

## Figures and Tables

**Fig. 13:**
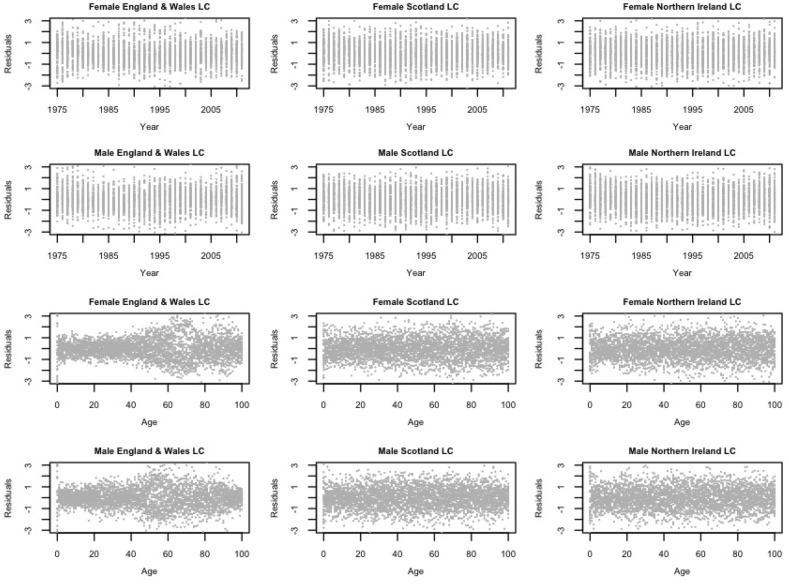
Residual plots by age and year of the LC model applied to the three countries and genders of UK

**Fig. 14:**
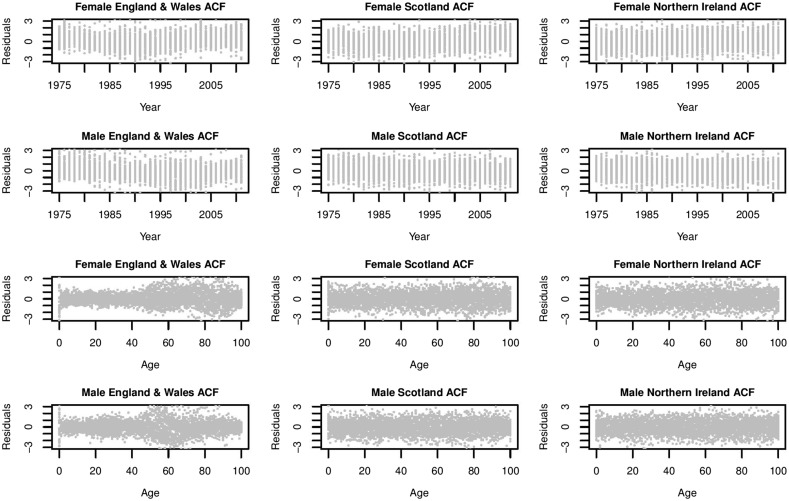
Residual plots by age and year of the 1-tier ACF model applied to each couple of genders within the three countries of UK

**Fig. 15:**
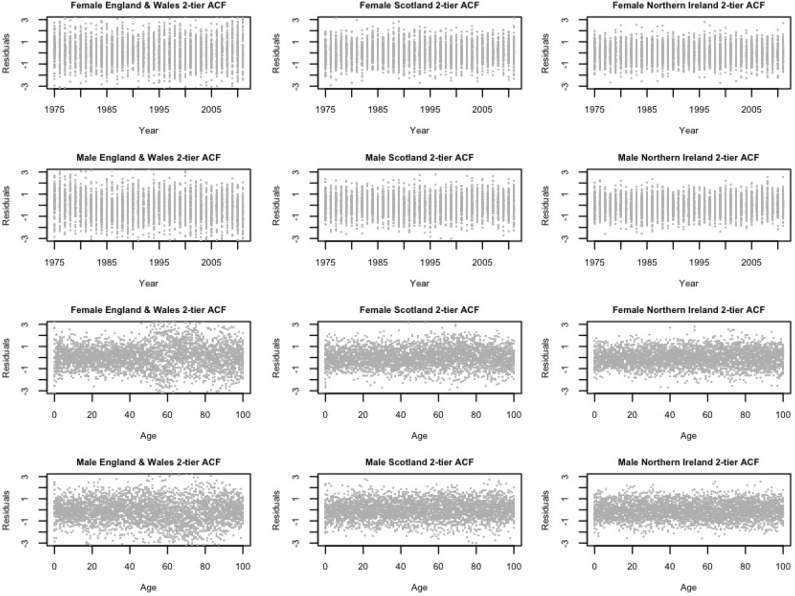
Residual plots by age and year of the 2-tier ACF model applied to the three countries of UK

**Fig. 16:**
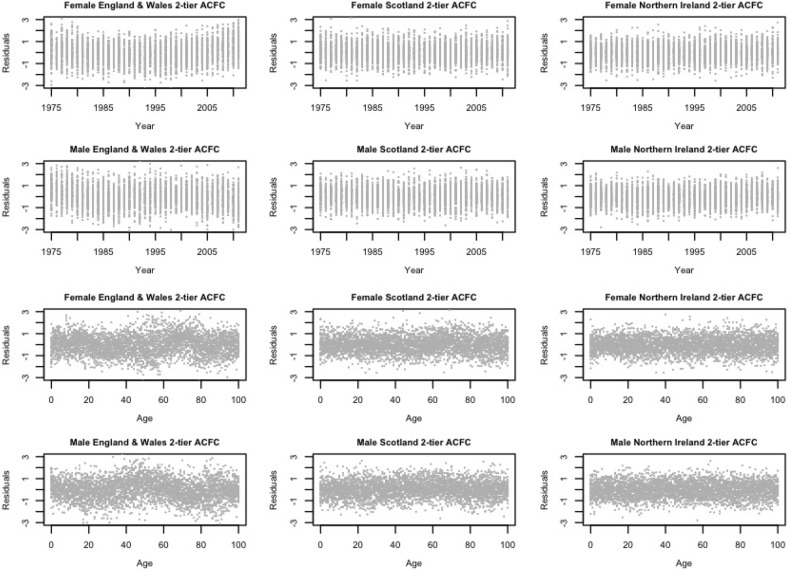
Residual plots by age and year of the 2-tier ACFC model applied to the three countries of UK

## Appendix A: Estimation algorithm

The parameters of the 2-tier ACF and 2-tier ACFC models are obtained by maximization of the log-likelihood (or minimization of the deviance), performed using the Newton–Raphson updating rule

$$\begin{aligned} \theta ^*=\theta -\frac{\partial \ell /\partial \theta }{\partial ^2 \ell /\partial \theta ^2}, \end{aligned}$$

where $\ell$ is the log-likelihood and $\theta$ represents any parameter to be fitted. The log-likelihood and deviance are defined by

$$\begin{aligned} \ell =&\sum _{x,t,i,j}\left[ d_{x,t,i,j} \log \widehat{d}_{x,t,i,j}-\widehat{d}_{x,t,i,j}-\log (d_{x,t,i,j}!)\right] \\ \text {deviance}=&\sum _{x,t,i,j}\left[ d_{x,t,i,j} \log \frac{d_{x,t,i,j}}{{\widehat{d}}_{x,t,i,j}}-d_{x,t,i,j}+\widehat{d}_{x,t,i,j}\right] . \end{aligned}$$

Recall that $d_{x,t,i,j}$ denotes the observed number of death for age *x*, year *t*, gender *i* and country *j*, and $\widehat{d}_{x,t,i,j}=E_{x,t,i,j}m_{x,t,i,j}$ is the corresponding theoretical number of deaths, where $m_{x,t,i,j}$ is given by either (4) or (9).

Adapting [3, 28], parameters are updated through the following stages and steps (Fig. 13).

- **STAGE 0.**
  - Step I. Initialise parameter values: $\widehat{a}_{x,i,j}$ as the mean of $\log m_{x,t,i,j}$ over *t* for all *x*, *i* and *j*, $\widehat{K}_t=\widehat{\kappa }_{t,i}=\widehat{k}_{t,i,j}=0$, $\widehat{B}_x=\widehat{b}_{x,i}=\widehat{b}_{x,i,j}=1/101$ and $g_{h,i}=0$ for all *t*, *x*, *i*, *j* and $h=t-x$.
  - **STAGE 1.** Fit $$\widehat{a}_{x,i,j}+\widehat{B}_x\widehat{K}_t$$.
  - Step II. Update $\widehat{a}_{x,i,j}^*=\widehat{a}_{x,i,j}+\sum _t (d_{x,t,i,j}-\widehat{d}_{x,t,i,j})/\sum _t\widehat{d}_{x,t,i,j}$ for all *x*, *i* and *j*, and recalculate $\widehat{d}_{x,t,i,j}$.
  - Step III. Update $\widehat{K}_t^*=\widehat{K}_t+\sum _{x,i,j}(d_{x,t,i,j}-\widehat{d}_{x,t,i,j})\, \widehat{B}_x/\sum _{x,i,j}\widehat{d}_{x,t,i,j}\,\widehat{B}_x^2$ for all *t*, adjusted by the constraint $\sum _tK_t=0$, and recalculate $\widehat{d}_{x,t,i,j}$.
  - Step IV. Update ${\widehat{B}}_x^*={\widehat{B}}_x+\sum _{t,i,j}(d_{x,t,i,j}-{\widehat{d}}_{x,t,i,j})\, {\widehat{K}}_t/\sum _{t,i,j}{\widehat{d}}_{x,t,i,j}\,{\widehat{K}}_t^2$ for all *x*, adjusted by the constraint $\sum _xB_x=1$, and recalculate ${\widehat{d}}_{x,t,i,j}$.
  - Step V. Repeat Steps II to IV till the deviance converges.
  - **STAGE 2.** Conditional on Stage 1, fit $\widehat{b}_{x,i}\widehat{\kappa }_{t,i}$.
  - Step VI. Update ${\widehat{\kappa }}_{t,i}^*={\widehat{\kappa }}_{t,i}+\sum _{x,j}( d_{x,t,i,j}-{\widehat{d}}_{x,t,i,j})\,{\widehat{b}}_{x,i}/\sum _{x,j}{\widehat{d}}_{x,t,i,j}\,{\widehat{b}}_{x,i}^2$ for all *t* and *i*, adjusted by the constraint $\sum _t\kappa _{t,i}=0$, and recalculate $\widehat{d}_{x,t,i,j}$.
  - Step VII. Update ${\widehat{b}}_{x,i}^{*}={\widehat{b}}_{x,i}+\sum _{t,j}( d_{x,t,i,j}-{\widehat{d}}_{x,t,i,j})\,{\widehat{\kappa }}_{t,i}/\sum _{t,j}\widehat{d}_{x,t,i,j}\,\widehat{\kappa }_{t,i}^2$ for all *x* and *i*, adjusted by the constraint $\sum _x b_{x,i}=1$, and recalculate $\widehat{d}_{x,t,i,j}$.
  - Step VIII. Repeat Steps VI to VII until the deviance converges.
  - **STAGE 3.** Conditional on Stages 1-2, fit $\widehat{g}_{h,i}$.
  - Step IX. Update
    $$\begin{aligned} {\widehat{g}}_{h,i}^{*}={\widehat{g}}_{h,i}+\frac{\sum _{x,t,t-x=h,j}\omega _{x,t}( d_{x,t,i,j}-{\widehat{d}}_{x,t,i,j})}{\sum _{x,t,t-x=h,j}\omega _{x,t}{\widehat{d}}_{x,t,i,j}} \end{aligned}$$
    for all *h* and *i*, adjusted by the constraint $\sum _{h=t-x} g_{h,i}=0$.^4^
  - Step X. Repeat Step IX until the deviance converges.
  - **STAGE 4.** Conditional on the Stages 1-3, fit $\widehat{b}_{x,i,j}\widehat{\kappa }_{t,i,j}$.
  - Step XI. Update $\widehat{\kappa }_{t,i,j}^*=\widehat{\kappa }_{t,i,j}+\sum _x ( d_{x,t,i,j}-\widehat{d}_{x,t,i,j})\,\widehat{b}_{x,i,j}/\sum _x\widehat{d}_{x,t,i,j}\,\widehat{b}_{x,i,j}^2$ for all *t*, *i* and *j*, adjusted by the constraint $\sum _t\kappa _{t,i,j}=0$, and recalculate $\widehat{d}_{x,t,i,j}$.
  - Step XII. Update $\widehat{b}_{x,i,j}^*=\widehat{b}_{x,i,j}+\sum _t ( d_{x,t,i,j}-\widehat{d}_{x,t,i,j})\,\widehat{\kappa }_{t,i,j}/\sum _t\widehat{d}_{x,t,i,j}\,\widehat{\kappa }_{t,i,j}^2$ for all *x*, *i* and *j*, and recalculate $\widehat{d}_{x,t,i,j}$.
  - Step XIII. Repeat Steps XI and XII until the deviance converges.

Stage 3 is skipped when fitting the 2-tier ACF model. The algorithm is designed in four stages so that, for each bilinear component, the period mortality indices and age sensitivities are fitted in a way that best explains the overall trend of an aggregated population, leaving any trends specific to a subpopulation to the next stage of the model fitting. This also ensures the convergence of the model under the stated identifiability constraints.

## Appendix B: Residuals by age and calendar year

See Figs. 14, 15 and 16.

## Footnotes

1 When fitting the independent LC models, death counts are specified through (3) coupled with (1) deprived of the error term, and completed with the first identifiability condition in (5). Finally, each period term is modelled as a random walk with drift.

2 When fitting the independent 1-tier ACF models, death counts are specified through (3) coupled with (2) deprived of the error term, and completed with the first two identifiability conditions in (5). Finally, each common period term is modelled as random walk with drift, while gender specific period terms are modelled as AR(1) time series.

3 These metrics are defined as follows:

$$\begin{aligned}&{\text {AIC}}=-2\widehat{\ell }+2 n_p,\qquad {\text {BIC}}= -2\widehat{\ell }+n_p \log n_d,\\&{\text {MAPE}}=\frac{1}{n_d} \sum _{x,t,i,j}\left| \frac{\widehat{d}_{x,t,i,j}-d_{x,t,i,j}}{d_{x,t,i,j}} \right| ,\\&{\text {ER}}=1-\frac{\sum_{x,t,i,j}\left[ d_{x,t,i,j}-\widehat{d}_{x,t,i,j}\right] ^2}{\sum _{x,t,i,j}[d_{x,t,i,j}-E_{x,t,i,j} \exp {(a_{x,i,j})}]^2}. \end{aligned}$$

Here, $n_p$ is the number of parameters net of the number of constraints, $n_d$ is the number of actual observations, $\widehat{\ell }$ is the maximized log-likelihood, $\widehat{m}_{x,t,i,j}$, $d_{x,t,i,j}$ and $\widehat{d}_{x,t,i,j}$ are respectively the fitted mortality rate, observed death count and fitted death count at age *x*, year *t*, gender *i* and country *j*. The fitted death counts are defined by $\widehat{d}_{x,t,i,j}=E_{x,t,i,j}\widehat{m}_{x,t,i,j}$, where the fitted mortality rate $\widehat{m}_{x,t,i,j}$ is given by (1) deprived of the error term for the independent LC models; by (2) deprived of the error term for the independent 1-tier ACF model; by (4) or (9) for the 2-tier ACF, respectively 2-tier ACFC models. In each case parameters are replaced with their estimates. See [4] for the definition and properties of AIC and BIC, and [28] for the use of MAPE and ER in the context of mortality forecasting.
